# Comparison of suture-bridge and independent double-row techniques for medium to massive posterosuperior cuff tears: a two-year retrospective study

**DOI:** 10.1186/s12891-023-06256-6

**Published:** 2023-02-28

**Authors:** Poyu Chen, Han-Wei Yeh, Yi Lu, Alvin Chao-Yu Chen, Yi-Sheng Chan, Alexandre Lädermann, Joe Chih-Hao Chiu

**Affiliations:** 1 Department of Orthopedic Surgery, Linkou Chang Gung Memorial Hospital, No.5, Fusing St., Gueishan District, Taoyuan City, 333 Taiwan; 2 Department of Occupational Therapy and Graduate Institute of Behavioral Sciences, College of Medicine, Chang Gung University, Taoyuan, Taiwan; 3 Linkou Chang Gung Memorial Hospital, Taoyuan, Taiwan; 4 Bone and Joint Research Center, Chang Gung Memorial Hospital, Linkou, Taiwan; 5 Comprehensive Sports Medicine Center (CSMC) Chang Gung Memorial Hospital, Taoyuan, Taiwan; 6 Department of Orthopedic Surgery, Keelung Chang Gung Memorial Hospital, Keelung, Taiwan; 7 Division of orthopedics and Trauma Surgery, Hôpital de la Tour, Meyrin, Switzerland; 8 Faculty of Medicine, University of Geneva, Geneva, Switzerland; 9 Orthopedics and Trauma Service, University Hospitals of Geneva, Geneva, Switzerland

**Keywords:** Rotator cuff tears, Suture-bridge, Independent double-row, Suture anchor, Surgical time

## Abstract

**Background:**

Transosseous-equivalent suture-bridge (TOE-SB) and independent double-row (IDR) repair techniques were developed to treat rotator cuff tears. The study was designed to prove that both TOE-SB and IDR techniques provided comparable clinical results and retear rate for medium to massive posterosuperior rotator cuff tears, while the surgical time and number of suture anchor used were less in the IDR group.

**Study design:**

Level of evidence: level III, Retrospective comparative study.

**Methods:**

Patients with medium to massive posterosuperior rotator cuff tears receiving arthroscopic TOE-SB and IDR between November 2016 to October 2019 were retrospectively enrolled. All patients were confirmed to have grade ≤ 2 fatty infiltration in the muscles of the torn tendons. Revision, concomitant subscapularis tear, acromiohumeral distance < 7 mm, glenohumeral osteoarthritis, partial repair, incomplete repair, partial thickness, or irreparable posterosuperior cuff tear were excluded. Surgical time, number of suture anchor used for the surgery, pre-operative, and post-operative clinical scores such as Constant-Murley score, subjective shoulder value (SSV), and visual analog scale (VAS) were compared. The retear rates between groups were evaluated by ultrasound.

**Results:**

Thirty-five IDR and thirty-five TOE-SB repairs were enrolled. The IDR technique required much fewer anchors than TOE-SB did to complete the cuff repair. The mean operation time in IDR and TOE-SB group were 86(18.23), and 114(18.7) (min), respectively (*P* <  0.01). The mean number of anchors used to complete the cuff repair was 2(0.17) in IDR and 3(0.61) in TOE-SB (*P* <  0.01). The Constant-Murley score improved from 34.9 ± 6.6 to 80.6 ± 9.4 in the IDR group, and 37.4 ± 6 to 81.9 ± 4.6 in the TOE-SB group (both *P* <  0.001). SSV improved from 24.6 ± 9.6 to 79.3 ± 10.6 in the IDR, and 27.9 ± 9 to 82.9 ± 6.9 in the TOE-SB group (both *P* <  0.001). VAS improved from 7.9 ± 0.6 to 1.5 ± 0.7 in the IDR, and 8 ± 0.5 to 1.3 ± 0.6 in the TOE-SB group (both *P* <  0.001) at final follow-up. No significant difference was found between the retear rates (14.3% in the IDR vs. 17.1% in the TOE-SB, respectively) in the 2-year follow-up.

**Conclusions:**

Both IDR and TOE-SB group provided comparable clinical results and retear rates for medium to massive posterosuperior rotator cuff tears. The surgical time and number of anchors used were less in the IDR group than in the TOE-SB group.

## Introduction

The optimized surgical technique for rotator cuff repair is to cover as much footprint as possible without undue tension. Double-row (DR) repair is believed to provide better footprint coverage and a lower retear rate than single-row (SR) repair [1]. A number of surgical techniques of DR repair have been described with promising clinical results [2–5]. Among them, transosseous-equivalent suture-bridge (TOE-SB) [4] and independent double-row (IDR) [5, 6] repair provide a similar retear rate for full-thickness rotator cuff tears around 9 to 29% [5, 7]. However, the number of anchors used for TOE-SB and IDR are not always identical. TOE-SB needs one or two suture-loaded anchors in the medial row and two to three knotless anchors in the lateral aspect of the greater tuberosity [8]. While IDR normally uses only two anchors, one implanted at the bone-cartilage junction, and the other one implanted at the lateral part of the greater tuberosity [5].

This study aimed to compare intraoperative data and associated number of suture anchor used in TOE-SB and IDR techniques. We hypothesized that both TOE-SB and IDR techniques provided similar clinical and radiological results regarding functional scores and retear rates. The surgical time and number of suture anchor used were less in the IDR group.

## Material and methods

### Patient selection and demographics

The study was approved by the Institutional Review Board at the hospital of the corresponding author, and written informed consent was obtained from all patients. All patients were collected and recruited in the Taoyuan Chang Gung Memorial Hospital database. From January 2017 to December 2020, patients 40 years of age and older who had failed nonoperative treatment for a full-thickness medium to massive posterosuperior rotator cuff tears were enrolled. The Tear size was intraoperatively measured using an arthroscopic probe and categorized as small, medium, large and massive by DeOrio et al. [9] All patients were confirmed to have grade ≤ 2 fatty degeneration in the muscles of the torn tendons on magnetic resonance imaging (MRI) [10]. Patients with a revision cuff, a concomitant subscapularis tear, acromiohumeral distance < 7 mm, partial repair, incomplete repair, partial thickness or irreparable posterosuperior cuff tear, loss of follow-up, a neurological, collagenous, circulatory disease, or degenerative joint diseases were excluded from the study [5]. All cuff repairs were performed by a single shoulder surgeon with more than 10 years of experience.

### Surgical procedure

#### Toe-SB

**Fig. 1:**
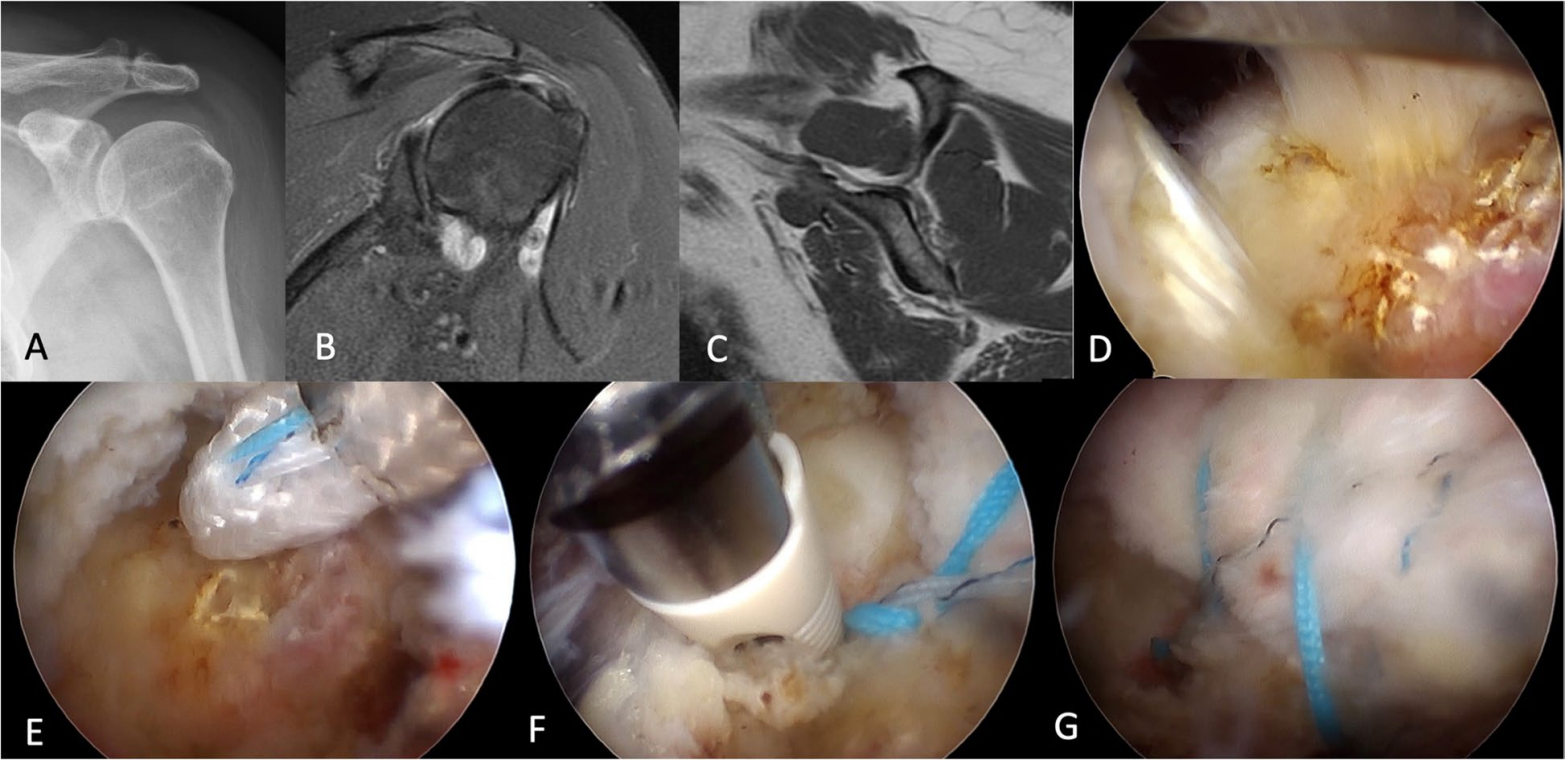
Surgical technique of TOE-SB repair. **A**-**C** A 65-year-old female patient had left supraspinatus tear without glenohumeral osteoarthritis and muscle fatty infiltration. **D** A crescent shape supraspinatus tear was confirmed during arthroscopy. **E** An all-suture anchor was inserted through anterolateral portal into the supraspinatus footprint as a medial row anchor. **F** Suture-bridge repair was carried out by fixating sutures from medial row anchor to the lateral aspect of the greater tuberosity with knotless anchors. **G** Final construct of the TOE-SB repair. TOE-SB, transosseous-equivalent suture-bridge

Diagnostic arthroscopy was first performed after debridement of inflamed tissue to determine the size and location of the torn cuff and the associated biceps lesion in a beach-chair position. Acromioplasty was not systemically performed but only when there was poor visualization during cuff repair or in type III acromion [11]. A high-speed bur was used to expose the cancellous bone. The torn rotator cuff was pulled with a grasper after adequate release, and a proper suture site was determined. Through a 5-mm incision, a metallic (TwinFix Ti 5.0, Smith & Nephew, Andover, MA), bioabsorbable (Healicoil Regenesorb 5.5, Smith & Nephew, Andover, MA), suture-based (CONMED Linvatec, Largo, Florida, USA), or PEEK suture-loaded anchor (Healicoil PK 5.5, Smith & Nephew, Andover, MA) was inserted through the anterolateral portal into the supraspinatus footprint 8 mm posterior to the bicipital as a medial row anchor, recreating anterior rotator cable [12]. One or two medial row anchors were used depending on the tear size. A suture was passed through the rotator cuff as proximally as possible using a Spectrum II suture hook (CONMED Linvatec, Largo, Florida, USA). The suture limb loaded onto the inserted suture anchor was passed using the shuttle relay technique. The suture limbs were tied in a horizontal mattress suture pattern. Suture-bridge repair was then carried out by fixating one limb from each anchor to the lateral aspect of the greater tuberosity using one or two knotless anchors, fully inserted at a perpendicular angle to the cortical surface of the humerus. Biceps tenotomy was done if more than 50% biceps tear was observed during the surgery, otherwise it was tenodesis with one suture limb of the medial row anchor. The complete surgical details are listed in Fig. 1.

#### IDR

**Fig. 2:**
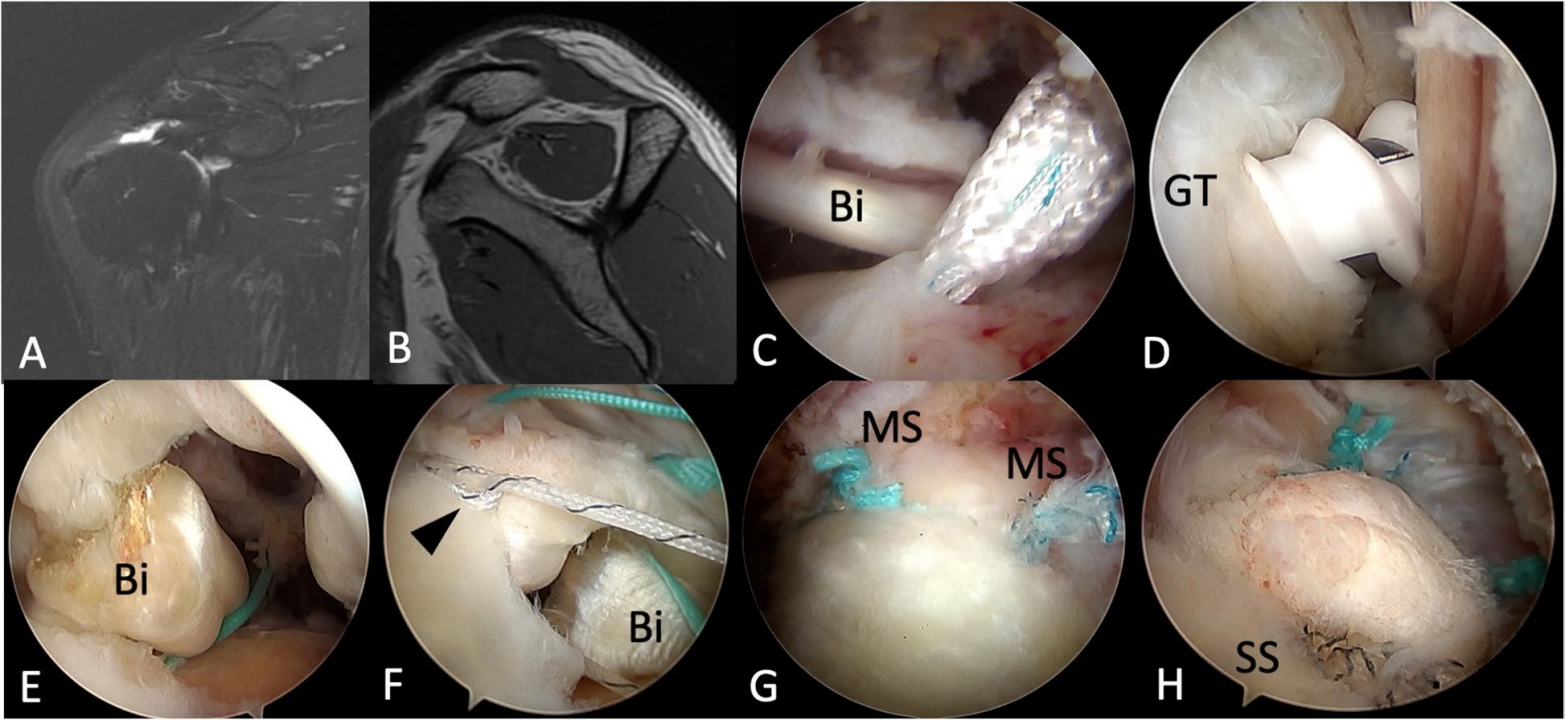
Surgical technique of IDR repair. **A**-**B** A 43-year-old male patient had right supraspinatus tear without glenohumeral osteoarthritis and grade 1 muscle fatty infiltration. **C** One triple-loaded all-suture anchor was implanted at the bone-cartilage junction 8 mm posterior to the bicipital groove as medial row anchor. **D** One suture-based anchor was implanted at the lateral part of the greater tuberosity. **E** Biceps long head was fixed with one limb from the medial row suture anchor. **F** Lasso-loop (arrowhead) can be made from the sutures of the lateral row anchor, increasing the grip force and compression area of posterosuperior cuff tears. **G** Final construct of the IDR repair. Two mattress sutures from medial row anchor. **H** Final construct of the IDR repair. Two simple lasso loop sutures from lateral row anchor. IDR, independent double-row; Bi, biceps long head; GT, greater tuberosity; MS, mattress sutures; SS, simple sutures

The diagnostic arthroscopy and tendon release were the same with the TOE-SB group. All repairs were made with double-loaded or triple-loaded suture anchors. Of which one was implanted at the bone-cartilage junction 8 mm posterior to the bicipital groove, and one was implanted at the lateral part of the greater tuberosity. Biceps tenotomy was done if more than 50% biceps tear was observed during the surgery, otherwise it was tenodesis with one suture limb of the medial row anchor. All sutures were managed by a Cuff Hook (Stryker, San Jose, CA, USA) suture manipulator. At the end of the intervention, all repairs were completely defined as repair up to the lateral end of the greater tuberosity footprint [13]. The complete surgical details are listed in Fig. 2.

### Rehabilitation protocol

Patients in both groups had identical rehabilitation protocol including wearing an abduction brace for 6 weeks following surgery. During the first 6 weeks, these patients only performed active hand, wrist, and elbow exercises, as well as active scapular retraction exercises. During the second 6-week period, active-assisted elevation in the plane of the scapula was initiated, followed by progression to active elevation. Formal physical therapy and a home exercise program continued 3-month after surgery [14].

### Main outcome measurements

The number of suture anchors used, surgical time determined by the time of timeout before the surgery and when the surgeon finished the surgery, and retear rates between TOE-SB and IDR groups were compared. The clinical outcomes were assessed pre-operatively and 2-year after operation by an independent orthopedic doctor. All patients were evaluated using (1) Constant-Murley shoulder score (CMS) [15], (2) Subjective shoulder value (SSV) [16], and (3) the visual analogue scale (VAS) pain scores. Tendon retear was evaluated by ultrasound examination performed in consensus by 2 independent observers: 1 musculoskeletal radiologist and 1 orthopedic surgeon different from the operating surgeon. Assessment of tendon healing was performed with an M-Turbo ultrasonography system (Fujifilm Sonosite, Tokyo, Japan). The ultrasound protocol consisted of (1) axial and longitudinal evaluation of the supraspinatus tendon, (2) axial and longitudinal evaluation of the infraspinatus tendon, and (3) presence of a subacromial or subdeltoid bursal fluid. When full footprint coverage was seen, the tendon was considered healed (type A); when the footprint was partially covered (type B) or uncovered (type C), the tendon was considered retear [17].

### Statistical analyses

We used chi-square test or independent *t*-test, as was appropriate to evaluate the homogeneity of baseline characteristics between IDR group and TOE-SB groups. The statistical software used was SPSS V.18.0 (SPSS, Chicago, IL, USA), and the significance was defined as *P* <  0.05. The sample size calculation was based on the primary outcome as CMS. The calculation showed that 24 cases in each group would provide adequate power to reject the null hypothesis (no difference between groups). The intra-rater reliability (intraclass correlation coefficient (ICC)) and the inter-rater reliability using unweighted Kappa to assess tendon healing were calculated [18].

## Results

**Fig. 3:**
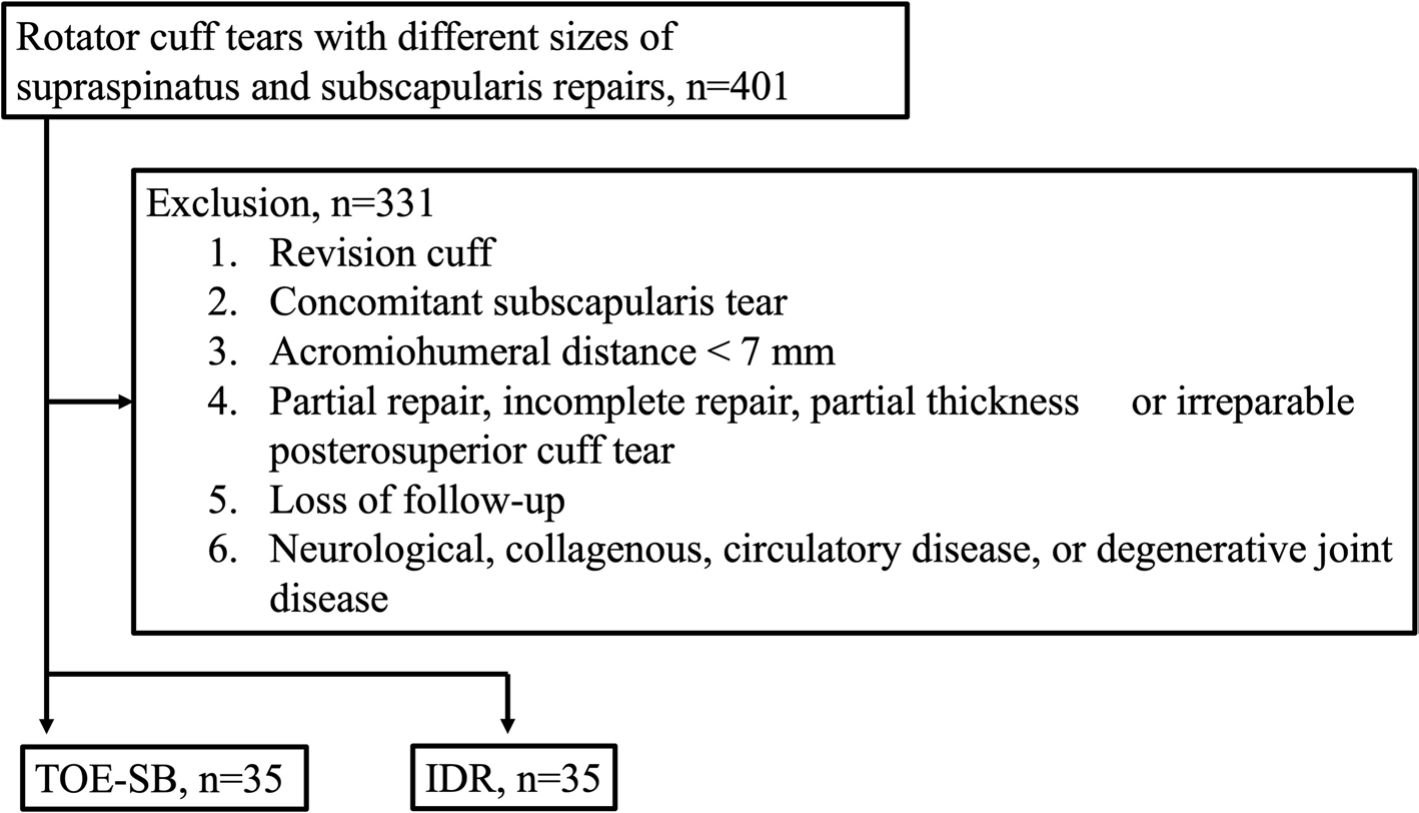
Study flow chart. IDR, independent double-row; TOE-SB, transosseous-equivalent suture-bridge

During the time, 401 rotator cuff tears with different sizes of supraspinatus and subscapularis repairs were done by the same surgeon. Among them, 70 patients (35 IDR and 35 TOE-SB repairs) were enrolled after the exclusion (Fig. 3). All patients fulfilled the 2-year follow-up. The average follow-up time was 25.6 ± 3.9 months. Preoperative demographic characteristics of both groups are summarized in Table 1. There was no significant difference between the two groups.

**Table 1:** Patient demographics in IDR and TOE-SB

	IDR	TOE-SB	*P*-value
**Sex, n (%)**			0.6145
Female	24 (68.57%)	22 (62.86%)	
Male	11 (31.43%)	13 (37.14%)	
**Age**	62.03 ± 9.63	59.94 ± 9.60	0.3673
**Dominant hand, Right:Left:both**	30:5:0	28:7:0	0.533
**Side**			0.179
Left	12 (34.29%)	7 (20.00%)	
Right	23 (65.71%)	28 (80.00%)	
**Size**			0.8443
Medium	16 (45.71%)	13 (37.14%)	
Large	15 (42.86%)	17 (48.57%)	
Massive	4 (11.43%)	5 (14.29%)	
**Tear Pattern**			0.5929
Crescent	30 (85.71%)	32 (91.43%)	
L-shape	2 (5.71%)	0 (0.00%)	
U-shape	3 (8.57%)	3 (8.57%)	
**Fatty Infiltration of Supraspinatus**			0.0266
0	6 (21.43%)	13 (54.17%)	
1	18 (64.29%)	7 (29.17%)	
2	4 (14.29%)	4 (16.67%)	

*IDR* independent double-row; *TOE-SB* transosseous-equivalent suture-bridge

### Clinical results

The pre-operative and 2-year follow-up CMS, SSV and VAS scores were similar between the two groups (Table 3). They were significantly improved 2 years after the operation compared with pre-operative condition (all p <  0.001) (Table 2). The minimal clinically important difference (MCID) for CMS in our study was 9.6 in IDR group (95% CI: 40.928–48.071, *P* <  0.001), and 5.9 in TOE-SB group (95% CI: 40.017–46.640, *P* <  0.001). The CMS achieved MCID at final follow-up in both groups in our study. The retear rates (14.3% in IDR vs. 17.1% in TOE-SB, respectively, *P* = 0.747) in the 2-year follow-up were comparable between the two groups. Both observers had excellent intra-rater reliability to assess tendon healing (ICC > 0.90.) The inter-rater reliability using unweighted Kappa was 0.869 in IDR group (*P* <  0.001), and 0.781 in TOE-SB group (*P* <  0.001), and achieved good level of agreement [18].

**Table 2:** Clinical results of IDR and SB groups

	Pre-op	Post-op 2Y	*P*-value
CMS			
IDR	34.9 ± 6.6	80.6 ± 9.4^a^	< 0.001
TOE-SB	37.4 ± 6	81.9 ± 4.6^a^	< 0.001
*P*-value	0.111	0.450	
SSV			
IDR	24.6 ± 9.6	79.3 ± 10.6^a^	< 0.001
TOE-SB	27.9 ± 9	82.9 ± 6.9^a^	< 0.001
*P*-value	0.142	0.097	
VAS			
IDR	7.9 ± 0.6	1.5 ± 0.7^a^	< 0.001
TOE-SB	8 ± 0.5	1.3 ± 0.6^a^	< 0.001
*P*-value	0.498	0.373	

*Pre-op* pre-operative; *Post-op* post-operative; *m* months; *y* years; *CMS* Constant-Murley Shoulder Score; *SSV* Subjective shoulder value; *VAS* visual analogue scale; *Y* years

### Number of anchors used and surgical time for both techniques

The IDR technique required 2(0.17) and the TOE-SB required 3(0.61) anchors to complete the cuff repair (*P* < 0.01). The mean operation time in IDR and TOE-SB group were 86(18.23), and 114(18.7) minutes to complete the surgery, respectively (*P* < 0.01) (Table 3).

**Table 3:** Total number of anchors used among IDR and SB groups

	IDR	TOE-SB	***P***-value
**Number of anchors used (SD)**	2(0.17)	3(0.61)	< 0.0001
2	34 (97.00%)	2 (5.71%)	
3	1 (3.00%)	16 (45.71%)	
4	0 (0.00%)	17 (48.57%)	
**Surgical time (SD, min)**	86 (18.23)	114 (18.07)	< 0.001

*SD* standard deviation; *IDR* independent double-row; *TOE-SB* transosseous-equivalent suture-bridge

## Discussion

In the present study, we proved both TOE-SB and IDR techniques provided similar retear rate for medium to massive posterosuperior rotator cuff tears. The surgical time and number of anchors used were less in the IDR group. This result can be further referenced for medical economic analysis to treat rotator cuff tears since suture anchors’ cost is a major burden for such surgery. It will be much more cost-effective to treat rotator cuff tears with fewer suture anchors as long as the procedure provides a similar ability to re-create the rotator cuff footprint coverage [19].

In 2016, Collin et al. [5] published a method of repairing 78 posterosuperior rotator cuff tears by an IDR technique. This technique yielded a high rate of tendon healing on the bone, with a lower complication rate than the traditional DR repair techniques. Only seven patients (9%) had retear in their group. Six tendons were avulsed from the bone, and one tear at the myotendinous junction [5]. The retear rate was comparable to the best results in patients receiving TOE-SB repairs with only 5–9% retears [7, 20]. In our study, we compared both IDR and TOE-SB groups and found the comparable clinical score and retear rates between groups, which encored the results of Collin et al. [5] Also, the surgical time and number of anchors used were less in the IDR group due to the design of the technique. Hence, IDR technique may provide a less expensive way to complete medium to massive posterosuperior rotator cuff repair with comparable clinical results.

In general, the TOE-SB technique needs one to two suture-based anchors put in the medial row and two to three knotless anchors in the greater tuberosity (lateral row) to provide better tendon-bone interface pressure than the conventional DR repair, which provides only point fixations [4]. However, there are still concerns regarding TOE-SB repairs, such as tear at the myotendinous junction, [21] weakness of “bridging self-reinforcing” assemblies with a single fixation point, [22] larger number of anchors, [23] dog-ear deformity, [24] and a large number of holes made in the tendon. In the IDR technique, two suture-based anchors are usually enough for a massive supraspinatus repair, making it economically advantageous over the TOE-SB technique [5]. Two lasso-loop sutures [25] can be made from lateral row anchor, increasing the grip force and compression area of large-to-massive posterosuperior cuff tears. On the other hand, the compression area provided by IDR can be determined by the point where the suturing device (e.g., Cuff Hook or suture lasso) penetrating the torn cuff. The technique also requires fewer holes in the cuff, which is advantageous given the instruments’ size. For example, when facing a large to massive posterosuperior cuff tear, we can pass the cuff in the most posterior, middle, and anterior part, providing as large compression area as possible by two medial mattress sutures and two lateral sutures. The other advantage of IDR is that the sutures from the medial anchor can also be used for tenodesis of the long head of the biceps or upper subscapularis repair. If a triple-loaded suture anchor is used in the medial row, the biceps can be served as a local tissue autograft for superior capsular reconstruction like Boutsiadis et al. proposed [26]. Since the retear rate of both techniques is similar, IDR may be an interesting alternative to TOE-SB when the cost of anchors and surgical time is considered in rotator cuff repairs.

There are still limitations in this study. First, the retrospective nature may have led to biases. Second, the types of suture anchors were not controlled. We used double-loaded metallic, absorbable, PEEK, all-suture, and triple-loaded all-suture anchors in both techniques. There were no anchor dislodgement or related complications to date during the follow-up period. Therefore, further subgroup analysis should be performed to see which type of anchor is the best for cuff repair. Third, the size and shape of cuff tears were not controlled. We only enrolled patients with medium to massive posterosuperior cuff tears because SR repair already works well for smaller tears [27]. Fourth, the biceps treatments were not controlled. We performed 27 biceps tenodesis and 6 tenotomies in the TOE-SB group, and 24 tenodesis and 7 tenotomies in the IDR group. The main objective of the current study was not to address the importance of the biceps treatments between groups, and most biceps tenodesis were done by fixing the biceps with sutures from medial row anchor.

## Conclusions

Both IDR and TOE-SB group provided comparable clinical results and retear rates for medium to massive posterosuperior rotator cuff tears. The surgical time and number of anchors used were less in the IDR group than in the TOE-SB group.

## Data Availability

The datasets used and/or analysed during the current study available from the corresponding author on reasonable request (Chih-Hao Chiu MD, PhD).
